# Accelerometer-measured sedentary behavior and risk of functional disability in older Japanese adults: a 9-year prospective cohort study

**DOI:** 10.1186/s12966-023-01490-6

**Published:** 2023-07-26

**Authors:** Tao Chen, Sanmei Chen, Takanori Honda, Hiro Kishimoto, Yu Nofuji, Kenji Narazaki

**Affiliations:** 1 Sports and Health Research Center, Department of Physical Education, Tongji University, 1239 Siping Road, Shanghai, 200-092 China; 2 Global Health Nursing, Department of Health Sciences, Graduate School of Biomedical and Health Sciences, Hiroshima University, 1-2-3 Kasumi, Minami Ward, Hiroshima, 734-8553 Japan; 3 Department of Epidemiology and Public Health, Graduate School of Medical Sciences, Kyushu University, 3-1-1 Maidashi, Higashi-Ku, Fukuoka, 812-8582 Japan; 4 Faculty of Arts and Science, Kyushu University, 744 Motooka Nishi-Ku, Fukuoka, 819-0395 Japan; 5 Research Team for Social Participation and Healthy Aging, Tokyo Metropolitan Institute for Geriatrics and Gerontology, 35-2 Sakae, Itabashi, Tokyo, 173-0015 Japan; 6 Center for Liberal Arts, Fukuoka Institute of Technology, 3-30-1 Wajiro-Higashi, Higashi-Ku, Fukuoka, 811-0295 Japan

**Keywords:** Sedentary time, Accelerometry, Primary prevention, Long-term care needs, Risk factors

## Abstract

**Background:**

The associations of sedentary time and patterns with functional disability among older adults remain unclear, and few studies have accounted for the co-dependency of sedentary behavior and physical activities when modeling sedentary behavior with risk of functional disability. We aimed to examine the associations between sedentary time and patterns and risk of incident functional disability, and assess whether replacing sedentary time with light physical activity (LPA) or moderate-to-vigorous intensity physical activity (MVPA) is associated with reduced risk of functional disability in community-dwelling older adults.

**Methods:**

A total of 1,687 Japanese adults aged ≥ 65 years without functional disability at baseline were prospectively followed-up for 9 years (2011–2020). Functional disability was ascertained using the national database of Japan’s Long-term Care Insurance System. Sedentary time and patterns, LPA, and MVPA were measured using a tri-axial accelerometer secured to participants’ waists.

**Results:**

During follow-up, 466 participants developed functional disability. Compared with the lowest quartile of total sedentary time, the multivariable-adjusted hazard ratios (95% confidence intervals) of functional disability for the second, third, and top quartiles were 1.21 (0.91‒1.62), 1.45 (1.10‒1.92), and 1.40 (1.05‒1.88) (*p* for trend = 0.01). After further adjusting for MVPA, total sedentary time was no longer significantly associated with the risk of functional disability (*p* for trend = 0.41). Replacing 10 min/day of sedentary time with the same amount of MVPA (but not LPA) was significantly associated with a 12% reduced risk of functional disability (hazard ratio [95% confidence interval]: 0.88 [0.84‒0.92]). No significant association was observed between sedentary bout length and functional disability.

**Conclusion:**

Higher levels of total sedentary time were associated with an increased risk of incident functional disability. However, this association was not independent of MVPA. Replacing sedentary time with MVPA, but not LPA, was associated with reduced risk of functional disability in older adults.

**Supplementary Information:**

The online version contains supplementary material available at 10.1186/s12966-023-01490-6.

## Introduction

Functional disability such as limitations on activities of daily living (ADL) and instrumental activities of daily living (IADL) among older adults causes increased acute care use [1], hospitalization [2], and death [3], resulting in considerable burdens on social, economic, and healthcare systems [4]. The number of older adults with functional disabilities is expected to increase substantially in the coming years as a result of population aging worldwide. There is therefore an urgent need to identify modifiable factors associated with the risk of onset of functional disability to inform public health recommendations and preventive strategies.

Compelling evidence has shown that engaging in moderate-to-vigorous intensity physical activity (MVPA) protects against functional disability [5–7]. Excessive time spent engaged in sedentary behavior has emerged as a risk factor for reduced muscle strength and power [8], chronic diseases [9, 10], and all-cause mortality in older adults [9, 11], even after accounting for MVPA, indicating that sedentary behavior is not simply physical inactivity (i.e., a lack of MVPA). More recently, sedentary time, particularly when accumulated in prolonged, uninterrupted bouts, has also been shown to be associated with health risks, including poorer physical function [12] and incidence of falls [13], which are established risk factors for functional disability in older adults. However, the associations of sedentary time and patterns with functional disability remain unclear. Two recent systematic reviews of objectively measured sedentary time showed that higher levels of total sedentary time are associated with increased odds of disability [14, 15]. However, most existing studies have been limited by cross-sectional designs [14, 15]. To the best of our knowledge, no prospective studies have examined whether prolonged patterns of sedentary behavior are associated with a higher risk of functional disability in older adults.

Although some evidence suggests that the health impact of sedentary time is independent of MVPA [9–11, 16], several recent studies have suggested that the beneficial effects of reducing sedentary time on health outcomes in older adults may be attributable to displacement by physical activities [17–20], including light physical activity (LPA) [19] with more profound effects of MVPA [17, 18, 20]. Therefore, the latest World Health Organization Guideline on Physical Activity and Sedentary Behavior states that “replacing sedentary time with physical activities at any intensity (including LPA) has health benefits,” in addition to highlighting the importance of reducing sedentary time [21]. However, few studies have accounted for the co-dependency of sedentary behavior and physical activities when modelling their association with the risk of functional disability [22]. In other words, it is unclear whether substituting sedentary time with LPA is adequate for reducing the risk of functional disability, or if MVPA is needed among older adults.

This study used a 9-year prospective design and aimed to investigate 1) whether total sedentary time or prolonged bouts of sedentary time are associated with the risk of incident functional disability in a cohort of community-dwelling older Japanese adults; and 2) whether replacing sedentary time with MVPA and/or LPA is associated with reduced risk of incident functional disability.

## Methods

### Participants

We used data from the Sasaguri Genkimon Study, which started in 2011 and is an ongoing community-based prospective study of residents aged ≥ 65 years in the town of Sasaguri, a suburb in Fukuoka Prefecture in southern Japan [23]. As of January 2011, the population of the Sasaguri town was 31,606, and the distributions of age, sex, education, and occupation in Sasaguri town were similar to that of the population in Japan as a whole (see Additional file 1), as we have previously described elsewhere [24]. A total of 4,979 residents aged 65 years or older met the inclusion criteria of the Sasaguri Genkimon Study of not being identified as needing to draw on long-term care insurance (LTCI). In Japan, LTCI is mandatory social insurance, with eligibility for benefits based strictly on physical and mental disability [25]. Every Japanese adult aged 65 or older is eligible to apply. After excluding 66 residents who had died or moved out of the town by the start of the baseline survey between May and August 2011, we contacted 4,913 eligible residents, and 53.5% of them agreed to participate (*n* = 2,629). For this analysis, we excluded nine participants who were certified as requiring LTCI before their baseline measurements. We further excluded 15 participants with a self-reported medical history of dementia or Parkinson’s disease, 858 participants without valid accelerometer data, and 60 participants without complete data on covariates, resulting in an analytic cohort of 1,687 participants (see Additional file 2). We reported this study by following the Strengthening the Reporting of Observational Studies in Epidemiology (STROBE) reporting guideline (see Additional file 3).

### Accelerometer data at baseline survey

At baseline participants were asked to wear a tri-axial accelerometer (Active Style Pro HJA-350IT, Omron Healthcare, Kyoto, Japan) on either side of their waist for 7 consecutive days when awake (removing it for any water-based activities) [7]. The accuracy of the activity intensity estimated by this accelerometer has been validated using the Douglas bag method [26, 27]. Non-wear time was computed using a SAS macro program provided by the National Cancer Institute [28], with modifications to fit the type of accelerometer used [29]. Non-wear time was defined as a consecutive period of no activity (i.e., estimated activity intensity < 1.0 metabolic equivalent [MET]) for at least 60 min, allowing for 2 min of activity when the intensity rose to 1.0 MET. Accelerometer data were considered valid when there were ≥ 4 valid wearing days (≥ 10 h of wear per day) [30].

The cutoff points for activity intensity used to define sedentary time, LPA, and MVPA were ≤ 1.5 METs, 1.6–2.9 METs, and ≥ 3 METs. A bout of sedentary behavior was defined as consecutive minutes of activity intensity ≤ 1.5 METs without interruption. Total sedentary time was defined as the total accumulated time spent in all bouts of sedentary behavior. Mean sedentary bout length was calculated as total sedentary time divided by the number of bouts. Higher bout length indicates more prolonged accumulation patterns, whereas lower bout length indicates interrupted patterns. The two measures of sedentary time, LPA, and MVPA were averaged across valid days and expressed in min/day.

### Measures of covariates at baseline survey

Information on age and sex was obtained from the municipality office. Years of formal education, living alone (yes or no), current smoking and drinking status (yes or no), and whether the participant had fallen in the previous year (yes or no) were obtained using a questionnaire. Body mass and height were measured using conventional scales, and body mass index (BMI) was calculated by dividing the body mass (kg) by height (m) squared (kg/m^2^). Multimorbidity was defined as the presence of two or more of 13 chronic diseases: hypertension, stroke, heart disease, diabetes mellitus, hyperlipidemia, respiratory disease, digestive disease, kidney disease, osteoarthritis or rheumatism, trauma fracture, cancer, ear disease, and eye disease. The presence of chronic diseases was self-reported via the questionnaire. Cognitive function was measured using the Japanese version of the Mini-Mental State Examination (MMSE). MMSE scores range from 0 to 30 points, with higher scores indicating better global cognitive function. Cognitive impairment was defined as an MMSE score < 24 points [31]. We used a single question of “Do you normally walk continuously for 15 min?” with an answer of “yes/no” to define low walking ability [32]. This question was obtained from the Kihon Checklist which was developed by the Ministry of Health, Labor and Welfare of Japan to identify older adults at high risk of functional disability [33, 34].

### Follow-up survey of incident functional disability

We prospectively followed up the participants from baseline until the newly onset of functional disability. The data on the incidence of functional disability, death, and moving out of the town during follow-up were provided by the municipal office of the Sasaguri town. Incident functional disability was ascertained using the national database of the LTCI system, which were provided by the municipal government office. In the LTCI system, certification of functional disability is determined using nationally standardized assessments of individual physical and mental status [25]. These standardized assessments include paralysis and limitation of joint movement, movement and balance, complex movement, conditions requiring special assistance, conditions requiring assistance with ADL/ IADL, communication and cognition, and behavioral problems [25]. A standardized scoring system is applied to calculate scores for physical and mental function and estimate the amount of time required for care in eight categories, including grooming and bathing, eating, toileting, transferring, assistance with IADL, behavioral problems, rehabilitation, and medical services [25]. A local certification committee of physicians, nurses, and other experts in health and social services then decides whether a given adult should be certified as needing care. Finally, the committee assigns care needs at one of seven levels (support levels 1–2; care levels 1–5) to each certified person. The type of available nursing care services depends on the level of care needs. For example, individuals at support level 1 can receive various nursing care services at home or in nursing facilities, such as home help services and home-visit bathing services [25]. The care need levels of LTCI certification are highly correlated with physical disability levels identified by the Barthel Index (Spearman’s ρ =  − 0.86), and moderately correlated with cognitive function levels assessed by the MMSE (Spearman’s ρ =  − 0.42) [35]. We defined the endpoint of functional disability as the onset of long-term care needs at the first level (support level 1) or above [7, 36, 37]. Follow-up duration was counted from the date of baseline survey until the date of being ascertained as functional disability, death, loss to follow-up (i.e., moving out of the town), or the end of follow-up (March 31, 2020), whichever came first.

### Statistical analysis

Given the strong correlation between total sedentary time and accelerometer wear time (*r* = 0.52), we corrected total sedentary time for accelerometer wear time by standardizing the total sedentary time to 16 h per day of wear time, using the residuals obtained by regressing total sedentary time on accelerometer wear time [11, 38]. The advantage of the residual method, as compared to adjusting for wear time as a covariate, is that the residuals from the regression represent the differences in each individual's actual sedentary time, effectively removing the variation caused by wear time, especially when the exposure variable was categorized [38, 39]. Mean bout duration was weakly correlated with accelerometer wear time (*r* = 0.09) and therefore was not corrected. Participants’ characteristics at baseline were summarized using means (standard deviation [SD]), medians (interquartile range [IQR]), or proportions across the quartiles of total sedentary time, as appropriate. Trends of characteristics across quartiles of total sedentary time were tested using the Jonckheere-Terpstra trend test for continuous variables and the Cochran–Armitage trend test for categorical variables.

Cox proportional hazard models were used to estimate hazard ratios (HRs) and 95% confidence interval (CI) of functional disability by quartiles of total sedentary time and mean sedentary bout duration. Model 1 was adjusted for sex and age. Model 2 was additionally adjusted for education, living alone, BMI, multimorbidity, fall experience in the past year, low walking ability, cognitive impairment, smoking, and drinking. Trends in functional disability risk across the quartiles of total sedentary time and mean sedentary bout duration were tested by assigning ordinal numbers (0, 1, 2, 3) to each quartile and treating the quartiles as a continuous variable.

To test the independent effect of sedentary time and patterns from MVPA [11, 16], we therefore additionally adjusted for MVPA (treated as a confounder) in a third model (Model 3). Because recent evidence also suggests that the effects of sedentary time depend on MVPA levels [40, 41], we examined the interactions of MVPA levels (< 150 and ≥ 150 min/week) with measures of sedentary behavior with adjustment for all the above-mentioned covariates. Interactions of sex and age groups (< 74 and ≥ 75 years) with sedentary time were also tested, to examine any potential modifying effects. The potential non-linear associations of total sedentary time and mean sedentary bout duration with functional disability were examined using restricted cubic splines with three knots placed at the 5th, 50th, and 95th percentiles [42]. The linearity of the association was examined using a likelihood ratio test.

We used the isotemporal substitution model to account for the codependence of sedentary time and physical activities, and to estimate the theoretical effects of replacing 10 min of sedentary time with equal time of LPA or MVPA [43]. For a complete isotemporal substitution analysis, we also examined replacing LPA or MVPA with other activities. The isotemporal substitution model has been used to assess the effects on health outcomes of alternating allocations of time between two behaviors while holding the total time constant [17–20, 43]. Specifically, to examine the effects of replacing sedentary time with other activities, LPA, MVPA, and wearing time were included in a single Cox model (each expressed continuously in 10-min units) after adjusting for the covariates in Model 2, with sedentary time (without wearing time correction) excluded from this model.

We conducted a sensitivity analysis by adjusting for accelerometer wear time as a covariate instead of the residual method used to correct for accelerometer wear time. To account for potential reverse-causation bias, we performed two sensitivity analyses by excluding 1) individuals who developed functional disability within the first 2 years of follow-up (n = 113), and 2) individuals who did not normally walk continuously for 15 min at baseline (n = 212). All statistical analyses used SAS version 9.4 (SAS Institute Inc., Cary, NC). The significance level was set at two-sided α = 0.05.

## Results

Participants’ mean age at baseline was 73.3 (SD 6.0) years and 62.2% of participants were women. The mean accelerometer wear time was 839.1 (SD 105.3) min/day. On average, participants spent 462.3 (SD 125.4) min/day (i.e., 7.7 [SD 2.1] hours/day) sedentary, 332.0 (SD 98.0) min/day (i.e., 5.5 [SD 1.6] hours/day) on LPA, and 44.8 (SD 34.4) min/day (i.e., 0.7 [SD 0.6] hours/day) on MVPA. The mean sedentary bout length was 8.2 min (SD 3.2). Table 1 shows the participants’ baseline characteristics by quartiles of total sedentary time. Participants who accumulated more sedentary time were more likely to be men and older, had higher mean values of BMI and higher rates of multimorbidity and low walking ability. They were more likely to be current smokers and drinkers.

**Table 1:** Baseline characteristics of the participants by quartile of total sedentary time (*n* = 1,687)^a^

	Quartile 1 (low)	Quartile 2	Quartile 3	Quartile 4 (high)	*P* for trend
	(*n* = 421)	(*n* = 422)	(*n* = 422)	(*n* = 422)	
Men, %	18.3	29.6	39.6	63.7	< .0001
Age, years	71.4 ± 5.2	72.9 ± 5.7	73.6 ± 6	75.4 ± 6.6	< .0001
Education, years	11.1 ± 2.2	11.1 ± 2.4	10.9 ± 2.4	11.4 ± 2.8	0.44
Living alone, %	10.7	14.9	13.0	14.0	0.28
BMI, kg/m^2^	22.5 ± 3	22.9 ± 3	23.5 ± 3.2	23.6 ± 3.2	< .0001
Multimorbidity, %	39.7	45.5	44.3	58.3	< .0001
Fall experience in the past year, %	20.9	18.3	16.6	21.6	0.97
Low walking ability, %	10.0	13.5	10.2	16.6	0.02
Cognitive impairment, %	5.0	3.8	5.9	6.6	0.15
Current smoker, %	5.0	5.2	8.5	10.4	0.0005
Current drinker, %	35.4	37.0	38.2	44.6	0.007
Accelerometer wear time, min/day	849.4 ± 96.4	829.9 ± 96.1	837.8 ± 108.7	839.5 ± 117.8	0.01
Total sedentary time, min/day	400.8 ± 48	500 ± 23	574.6 ± 20.3	673.4 ± 51.2	< .0001
Mean sedentary bout length, min/day	5.6 ± 1.2	6.9 ± 1.4	8.5 ± 1.8	11.6 ± 3.9	< .0001
LPA, min/day	444.6 ± 58.7	363.3 ± 45.8	302.3 ± 46.8	218.2 ± 56.8	< .0001
MVPA, min/day	72.6 ± 40.6	47.3 ± 27.2	36.8 ± 25.3	22.7 ± 19.3	< .0001

Continuous variables are shown as mean ± standard deviation. The quartile cut-off points of total sedentary time were 385.1, 462.4, and 536.3 min/day

*BMI* body mass index, *LPA* light physical activity, *MVPA* moderate-to-vigorous physical activity

^a^Values of total sedentary time were corrected for accelerometer wear time by standardizing the total sedentary time to 16 h per day of accelerometer wear time, using the residuals obtained by regressing total sedentary time on accelerometer wear time

During a median follow-up of 8.8 (IQR 6.0–8.8) years and 12,185 person-years, 466 participants developed functional disability, 129 died before experiencing any functional disability, and 85 moved out of town. Table 2 shows the associations between the measures of sedentary behavior and incidence of functional disability. There were direct associations across the quartiles of total sedentary time, with a greater risk of functional disability in higher quartiles (Model 2, *P* for trend = 0.01). The mean sedentary bout length was not significantly associated with the risk of functional disability in either model. After further adjusting for MVPA, total sedentary time was no longer significantly associated with the risk of functional disability (Model 3, *P* for trend = 0.41). The association of total sedentary time and mean sedentary bout length with risk of functional disability did not vary by MVPA levels, age groups, or sex (all *P* values for interactions > 0.05).

**Table 2:** Hazard ratios for the risk of functional disability by total sedentary time and mean sedentary bout duration quartiles (*n* = 1,687)^a^

	No. of events/participants	Incidence rate per 1000 person-years	Model 1		Model 2		Model 3	
			HR (95% CI)	*P* value	HR (95%CI)	*P* value	HR (95% CI)	*P* value
Total sedentary time								
Quartile 1 (low)	87/421	26.4	1.00		1.00		1.00	
Quartile 2	104/422	33.5	1.20 (0.90‒1.60)	0.22	1.21 (0.91‒1.62)	0.19	0.94 (0.70‒1.27)	0.69
Quartile 3	133/422	44.9	1.42 (1.08–1.87)	0.01	1.45 (1.10‒1.92)	0.01	1.02 (0.76‒1.37)	0.90
Quartile 4 (high)	142/422	50.3	1.41 (1.06‒1.87)	0.02	1.40 (1.05‒1.88)	0.02	0.85 (0.61‒1.18)	0.33
*P* for trend				0.01		0.01		0.41
Mean sedentary bout length								
Quartile 1 (low)	96/421	30.0	1.00		1.00		1.00	
Quartile 2	106/422	34.1	1.06 (0.81‒1.40)	0.66	1.07 (0.81‒1.42)	0.63	0.95 (0.71‒1.25)	0.70
Quartile 3	117/422	38.7	1.06 (0.80‒1.39)	0.70	1.10 (0.83‒1.44)	0.51	0.92 (0.70‒1.22)	0.58
Quartile 4 (high)	147/422	51.5	1.22 (0.93‒1.60)	0.15	1.22 (0.93‒1.60)	0.15	0.93 (0.70‒1.23)	0.60
*P* for trend				0.16		0.15		0.61

Model 1 was adjusted for age and sex

Model 2 was additionally adjusted for education, living alone, body mass index, multimorbidity, having a fall in the past year, low walking ability, cognitive impairment, smoking, and drinking

Model 3 was additionally adjusted for moderate-to-vigorous physical activity

*CI* confidence interval, *HR* hazard ratio

^a^Values of total sedentary time were corrected for accelerometer wear time by standardizing the total sedentary time to 16 h per day of accelerometer wear time, using the residuals obtained by regressing total sedentary time on accelerometer wear time. The quartile cut-off points were 460.1, 537.4, and 611.3 min/day for total sedentary time and 6.1, 7.5, and 9.4 min/day for mean sedentary bout duration

Cubic spline analysis showed that total sedentary time was associated with the risk of functional disability in a linear dose–response manner (Model 2, *P* for linear = 0.01; *P* for nonlinear = 0.22) (see Additional file 4, Figure A). Additional adjustment for MVPA attenuated the association (Model 3, *P* for linear = 0.34; *P* for nonlinear = 0.73) (see Additional file 4, Figure B). No significant association was observed for mean sedentary bout length (Model 2, *P* for linear = 0.22; *P* for nonlinear = 0.95).

In the isotemporal substitution models, replacing 10 min/day of total sedentary time with 10 min/day of LPA was associated with an HR (95% CI) of 1.00 (0.99–1.02) for functional disability. However, the replacement with 10 min of MVPA was significantly associated with a 12% lower risk of functional disability (HR [95% CI]: 0.88 [0.84–0.92]; Table 3).

**Table 3:** Multivariable-adjusted hazard ratios of functional disability associated with 10-min/day changes in time spent sedentary and in LPA and MVPA (*n* = 1,687)

	With sedentary time	With LPA	with MVPA
	HR (95% CI)	HR (95% CI)	HR (95% CI)
Replace sedentary time	–	1.00 (0.99–1.02)	0.88 (0.84–0.92)
Replace LPA	1.00 (0.99–1.01)	–	0.87 (0.83–0.92)
Replace MVPA	1.14 (1.09–1.20)	1.15 (1.09–1.21)	–

*CI* confidence interval, *HR* hazard ratio, *LPA* light physical activity, *MVPA* moderate-to-vigorous physical activity

Models were adjusted for sex, age, living alone, body mass index, multimorbidity, having a fall in the previous year, low walking ability, cognitive impairment, smoking, drinking, and accelerometer wear time

In sensitivity analyses, adjusting for accelerometer wear time as a covariate showed similar results with that of using the residual method to correct for variation in accelerometer wear time (see Additional file 5). Exclusion of participants who were certified as having functional disability in the first 2 years of follow-up resulted in a slight attenuation in the associations between total sedentary time and risk of functional disability (see Additional file 6), and the isotemporal substitution results were minimally changed (see Additional file 7). Exclusion of participants who did not normally walk continuously for 15 min at baseline did not materially change either (see Additional files 8 and 9).

## Discussion

In this prospective cohort study of older Japanese adults, higher levels of total sedentary time were associated with an increased risk of functional disability, but the risk was abolished when adjusting for MVPA. Replacing sedentary time with MVPA, but not LPA, was associated with a reduced risk of functional disability. Our findings suggest that reducing daily sedentary time alone, without increasing MVPA, may be inadequate for reducing the risk of functional disability in older adults. To our knowledge, the present study is one of the few to model functional disability risk by replacing sedentary time with LPA and MVPA.

This study reinforces previous cross-sectional studies of IADL or ADL disability [14, 15] showing an association between longer accelerometer-measured sedentary time with increased incidence of functional disability over a 9-year period. A 2-year prospective study in older men using accelerometer-measured sedentary time reported similar associations with risk of in both ADL and IADL disability, but it did not control for physical activity, leaving the independence of these associations from MVPA uncertain [44]. Two prospective cohort studies addressed this issue by employing self-reported sedentary time but revealed contrasting results: one observed an increased risk of mobility disability in older women, even after adjusting for physical activity [45], while another found that the association between sedentary time and functional limitation became negligible when considering LPA and MVPA [46]. Similarly, our study showed that the association between sedentary time and functional disability was not statistically independent of MVPA. It is evident that sedentary time and physical activity are intertwined, with an increase in one often resulting in a decrease in the other [18]. Therefore, our findings, combined with existing evidence, suggest that the adverse effects of excess sedentary time on long-term health outcomes such as function disability may be, at least in part, due to the displacement of physical activity, particularly MVPA [17–19].

In our isotemporal substitution analysis, replacing 10 min of sedentary time with MVPA was significantly associated with a 12% lower risk of functional disability. However, substituting with LPA did not reduce the risk. Our findings align with a previous prospective study in Japan, which reported a 14% risk reduction in functional disability over 2 years when replacing 10 min of sedentary time with MVPA, but not LPA [22]. In our prior study within the same cohort, we found a clear dose–response association between MVPA and functional disability, while the role of LPA remains less conclusive [7]. This suggests that higher intensity activity may be needed to prevent functional disability in older adults. In agreement with our findings, recent studies consistently showed that substituting sedentary time with MVPA, but not LPA, was associated with favorable functional outcomes in middle-aged and older populations [17, 47]. Specifically, such substitution with MVPA, but not LPA, was associated with reduced prevalence of sarcopenia, and better physical fitness [17], as well as better cognitive performance [47]. Our study extends previous work, indicating that solely reducing sedentary time or replacing sedentary time with LPA without increasing MVPA might not be sufficient to maintain functional independence.

The major strengths of our study include its prospective cohort design, the population-based relatively large sample, and the objective measure of time and patterns of sedentary behaviors, MVPA, and LPA using tri-axial accelerometers. Objective measures are more precise than subjective self-reported measures and involve less recall bias. The current study involved several limitations that should be considered. First, the accelerometer could not distinguish between standing and sitting. Thus, quiet standing could be misclassified as sedentary time, which may have led to an overestimation of sedentary time, consequently resulting in an underestimation of the association between sedentary time and risk of functional disability. In addition, the built-in algorithm of the accelerometer used in the present study was developed and validated in adults (aged 20–59 years) and has been shown to underestimate the actual MET of activities among older adults, particularly for more intense activity of 3 MET or higher [48]. Therefore, the amount of MVPA could have been underestimated, and the observed effects of replacing sedentary time with MVPA may have also been underestimated. Second, sedentary behavior and covariates were measured only at baseline and their changes during follow-up were not considered. This may have biased our results toward the null, resulting in an underestimation of the observed associations. Third, residual confounding is likely, although we accounted for a wide range of confounding factors. Fourth, we cannot rule out reverse causation because of uncertified functional disability at baseline. Participants who were vulnerable in the years immediately preceding the onset of functional disability may have been more likely to be sedentary at baseline. However, we controlled for low walking ability and other major confounders at baseline to minimize this potential bias. Sensitivity analyses by excluding participants that were certified as having functional disability in the first 2 years of follow-up and those who did not normally walk continuously for 15 min at baseline showed that the results for the isotemporal substitution analysis were minimally altered. Fifth, the exclusion of participants from the final sample mainly resulted from inadequate accelerometer wear time or days. Participants in the final sample may therefore have been more physically active than the general population (Additional file 2), which could have caused an underestimation of the associations. Finally, we urge caution in generalizing the current findings, because the study was undertaken in a single town in Japan.

## Conclusion

This study suggests that higher levels of total sedentary time were associated with an increased risk of incident functional disability; however, this association was not independent of MVPA. Replacing sedentary time with MVPA, but not LPA, was associated with a reduced risk of functional disability. While recent physical activity guidelines recommend that older adults should limit the amount of time spent being sedentary, our findings suggest that promotion of MVPA should still be prioritized to reduce the risk of functional disability.

## Supplementary Information

**Additional file 1. **The distribution of age, gender, and education in Sasaguri Town and in Japan as a whole.

**Additional file 2. **Characteristics of included participants versus excluded participants due to without valid accelerometer data in present study.

**Additional file 3. **STROBE Statement—Checklist of items that should be included in reports of cohort studies.

**Additional file 4. **Restricted cubic splines for the association between total sedentary time and risk of functional disability.

**Additional file 5. **Hazard ratios for the risk of functional disability by total sedentary time and mean sedentary bout duration quartiles adjusting for the accelerometer wear time directly.

**Additional file 6. **Hazard ratios for the risk of functional disability by total sedentary time and mean sedentary bout duration quartiles after excluding participants certified as functional disability in the first two year of follow-up (*n* = 1,574).

**Additional file 7. **Multivariable-adjusted hazard ratios of functional disability associated with 10-min changes in time spent in sedentary, LPA, and MVPA after excluding participants certified as functional disability in the first two year of follow-up (*n* = 1,574).

**Additional file 8. **Hazard ratios for the risk of functional disability by total sedentary time and mean sedentary bout duration quartiles after excluding participants who did not normally walk continuously for 15 min at baseline (*n* = 1,475).

**Additional file 9. **Multivariable adjusted hazard ratios of functional disability associated with 10-min changes in time spent in sedentary, LPA, and MVPA after excluding participants who did not normally walk continuously for 15 min at baseline (*n* = 1,475).

## Data Availability

The datasets used and/or analyzed during this study are available from the corresponding author on reasonable request.

## Abbreviations

ADL: Activities of daily living
BMI: Body mass index
CI: Confidence interval
HR: Hazard ratio
IADL: Instrumental activities of daily living
IQR: Interquartile range
LPA: Light physical activity
LTCI: Long-term care insurance
MET: Metabolic equivalent
MMSE: Mini-Mental State Examination
MVPA: Moderate-to-vigorous intensity physical activity
SD: Standard deviation
