# Supplementary material for: Accelerometer-measured sedentary behavior and risk of functional disability in older Japanese adults: a 9-year prospective cohort study
Source: Int J Behav Nutr Phys Act. 2023 Jul 26;20:91. doi: 10.1186/s12966-023-01490-6 (PMC10369703; doi:10.1186/s12966-023-01490-6)
Supplement: Supplementary file 1 — Additional file 1. The distribution of age, gender, and education in Sasaguri Town and in Japan as a whole. [file 12966_2023_1490_MOESM1_ESM.docx]

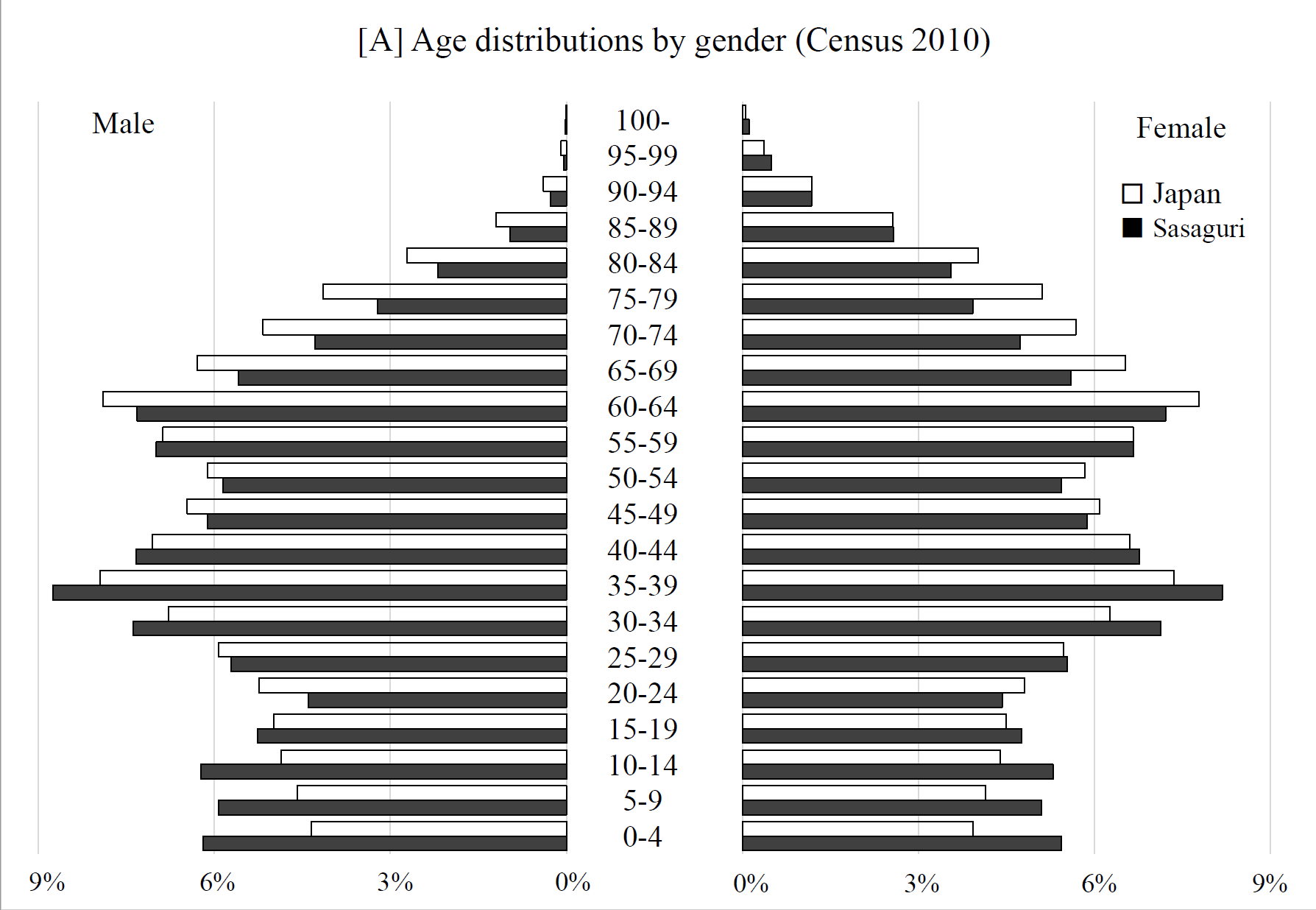


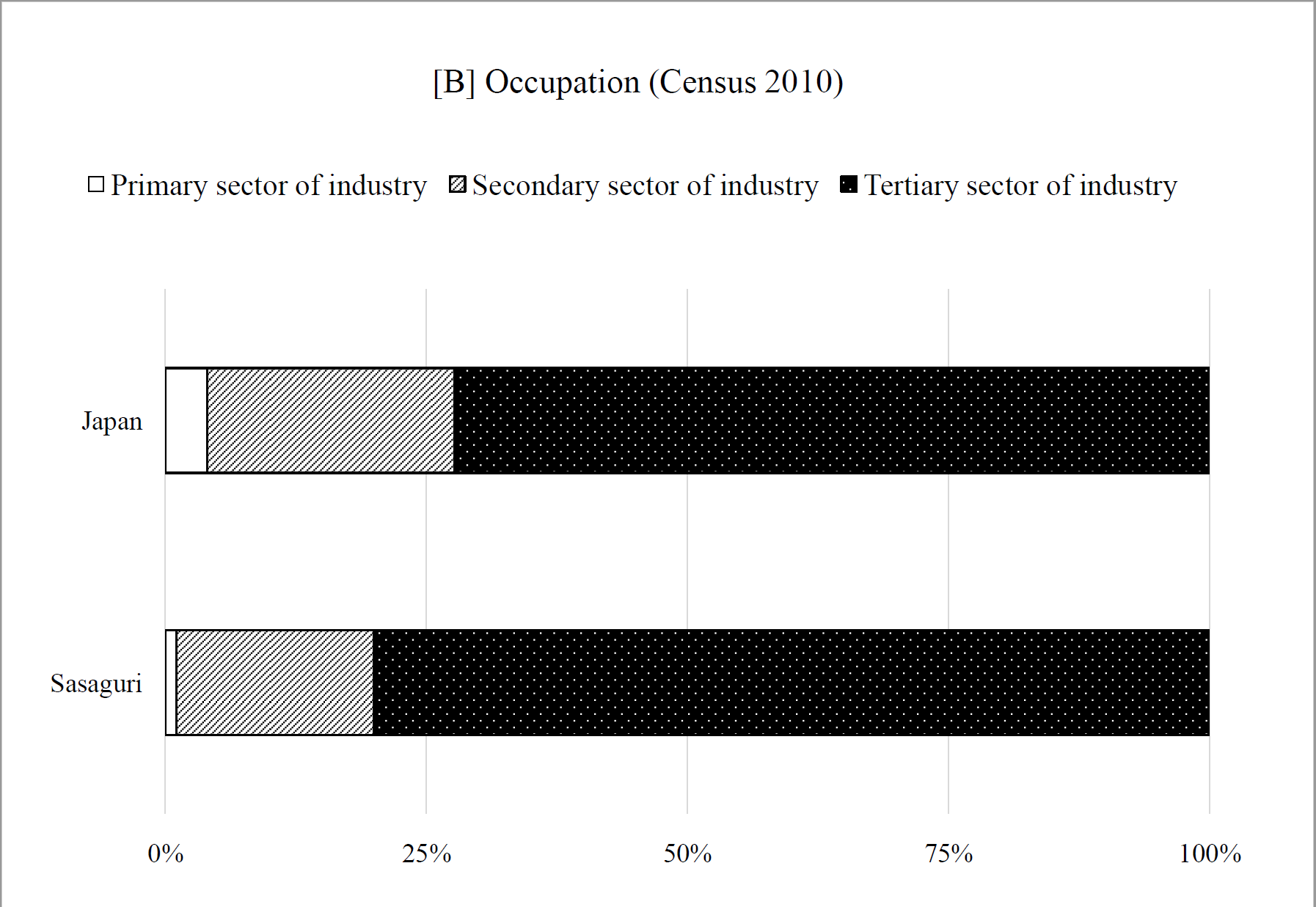

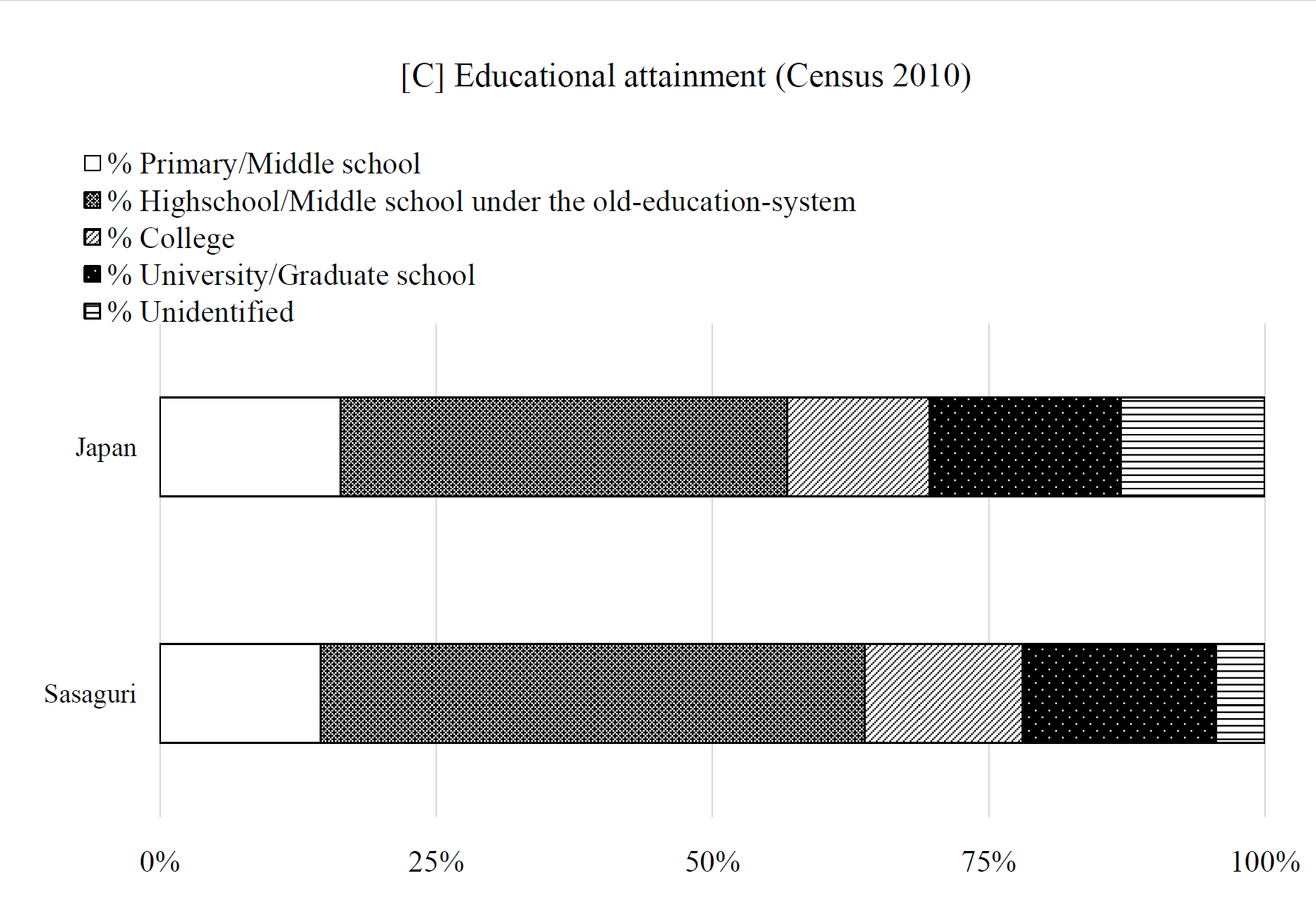


**Additional File 1.** The distribution of age, gender, and education in Sasaguri Town and in Japan as a whole
