# Supplementary material for: Accelerometer-measured sedentary behavior and risk of functional disability in older Japanese adults: a 9-year prospective cohort study
Source: Int J Behav Nutr Phys Act. 2023 Jul 26;20:91. doi: 10.1186/s12966-023-01490-6 (PMC10369703; doi:10.1186/s12966-023-01490-6)
Supplement: Supplementary file 2 — Additional file 2. Characteristics of included participants versus excluded participants due to without valid accelerometer data in present study. [file 12966_2023_1490_MOESM2_ESM.docx]

| **Additional File 2.** Characteristics of included participants versus excluded participants due to without valid accelerometer data in present study | | | | |
| --- | --- | --- | --- | --- |
|  | No. of participants with missing data | Included  (n = 1,687) | Excluded participants without valid accelerometer data  (n = 858) | *P* value^a^ |
| Men, n (%) | 0 | 638 (37.8) | 472 (55) | <.0001 |
| Age, years | 0 | 73.3 ± 6 | 73.6 ± 6.4 | 0.29 |
| Education, years | 32 | 11.1 ± 2.4 | 11.1 ± 2.7 | 0.68 |
| Living alone, n (%) | 19 | 222 (13.2) | 92 (11) | 0.12 |
| BMI, kg/m^2^ | 54 | 23.1 ± 3.1 | 22.9 ± 3.3 | 0.12 |
| Multimorbidity, n (%) | 0 | 792 (47) | 361 (42.1) | 0.02 |
| Fall experience in the past year, n (%) | 30 | 326 (19.3) | 192 (23.2) | 0.02 |
| Low walking ability, n (%) | 20 | 212 (12.6) | 132 (15.8) | 0.03 |
| Cognitive impairment, n (%) | 479 | 90 (5.3) | 58 (15.3) | <.0001 |
| Current smoker, n (%) | 25 | 123 (7.3) | 125 (15) | <.0001 |
| Current drinker, n (%) | 25 | 654 (38.8) | 384 (46.1) | <.001 |

*BMI*, body mass index.

Continuous variables are represented as mean ± SD.

^a^ Statistical significance based on chi-square tests or t-tests, as appropriate.
