# Supplementary material for: Accelerometer-measured sedentary behavior and risk of functional disability in older Japanese adults: a 9-year prospective cohort study
Source: Int J Behav Nutr Phys Act. 2023 Jul 26;20:91. doi: 10.1186/s12966-023-01490-6 (PMC10369703; doi:10.1186/s12966-023-01490-6)
Supplement: Supplementary file 4 — Additional file 4. Restricted cubic splines for the association between total sedentary time and risk of functional disability. [file 12966_2023_1490_MOESM4_ESM.pptx]

## Slide 1
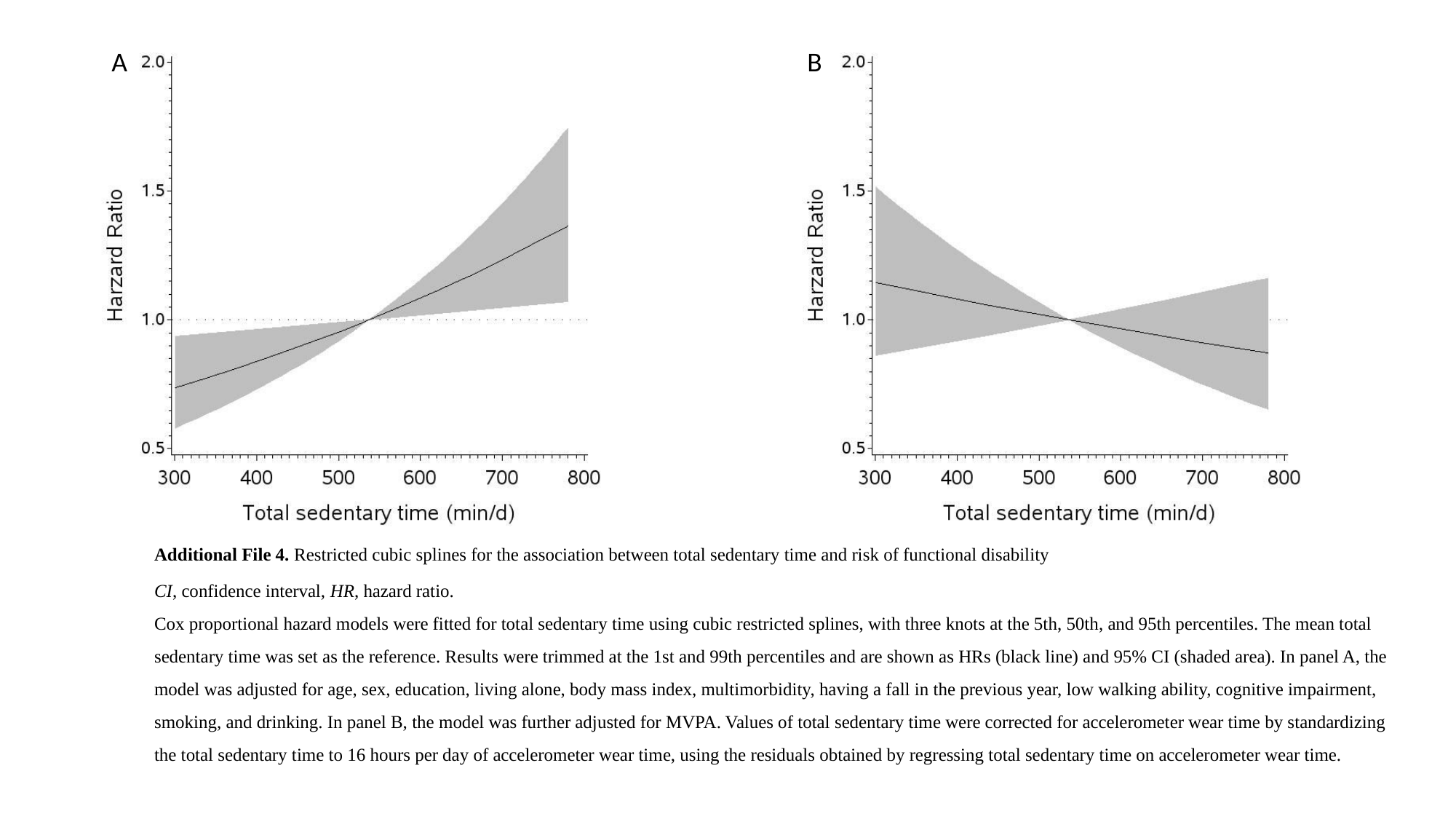

A
B
Additional File 4. Restricted cubic splines for the association between total sedentary time and risk of functional disability
CI, confidence interval, HR, hazard ratio.
Cox proportional hazard models were fitted for total sedentary time using cubic restricted splines, with three knots at the 5th, 50th, and 95th percentiles. The mean total sedentary time was set as the reference. Results were trimmed at the 1st and 99th percentiles and are shown as HRs (black line) and 95% CI (shaded area). In panel A, the model was adjusted for age, sex, education, living alone, body mass index, multimorbidity, having a fall in the previous year, low walking ability, cognitive impairment, smoking, and drinking. In panel B, the model was further adjusted for MVPA. Values of total sedentary time were corrected for accelerometer wear time by standardizing the total sedentary time to 16 hours per day of accelerometer wear time, using the residuals obtained by regressing total sedentary time on accelerometer wear time.
