# Supplementary material for: Accelerometer-measured sedentary behavior and risk of functional disability in older Japanese adults: a 9-year prospective cohort study
Source: Int J Behav Nutr Phys Act. 2023 Jul 26;20:91. doi: 10.1186/s12966-023-01490-6 (PMC10369703; doi:10.1186/s12966-023-01490-6)
Supplement: Supplementary file 5 — Additional file 5. Hazard ratios for the risk of functional disability by total sedentary time and mean sedentary bout duration quartiles adjusting for the accelerometer wear time directly. [file 12966_2023_1490_MOESM5_ESM.docx]

| **Additional File 5.** Hazard ratios for the risk of functional disability by total sedentary time and mean sedentary bout duration quartiles adjusting for the accelerometer wear time directly | | | | | | | | | | |
| --- | --- | --- | --- | --- | --- | --- | --- | --- | --- | --- |
|  | No. of events/  participants | Incidence rate per 1000  person-years | Model 1 | |  | Model 2 | |  | Model 3 | |
|  |  |  | HR (95% CI) | *P* value |  | HR (95% CI) | *P* value |  | HR (95% CI) | *P* value |
| Total sedentary time |  |  |  |  |  |  |  |  |  |  |
| Quartile 1 (low) | 97/421 | 30.4 | 1.00 |  |  | 1.00 |  |  | 1.00 |  |
| Quartile 2 | 120/422 | 39.5 | 1.27 (0.97–1.67) | 0.09 |  | 1.28 (0.98–1.69) | 0.07 |  | 1.00 (0.75–1.33) | 0.99 |
| Quartile 3 | 120/422 | 39.1 | 1.17 (0.88–1.54) | 0.27 |  | 1.20 (0.90–1.59) | 0.22 |  | 0.82 (0.61–1.12) | 0.21 |
| Quartile 4 (high) | 129/422 | 44.7 | 1.45 (1.07–1.97) | 0.02 |  | 1.44 (1.06–1.97) | 0.02 |  | 0.85 (0.60–1.21) | 0.36 |
| *P* for trend |  |  |  | 0.04 |  |  | 0.047 |  |  | 0.21 |
| Mean sedentary bout length | |  |  |  |  |  |  |  |  |  |
| Quartile 1 (low) | 96/421 | 30.0 | 1.00 |  |  | 1.00 |  |  | 1.00 |  |
| Quartile 2 | 106/422 | 34.1 | 1.08 (0.82–1.43) | 0.58 |  | 1.09 (0.82–1.44) | 0.54 |  | 0.96 (0.72–1.27) | 0.77 |
| Quartile 3 | 117/422 | 38.7 | 1.09 (0.83–1.44) | 0.52 |  | 1.14 (0.86–1.50) | 0.36 |  | 0.95 (0.71–1.26) | 0.71 |
| Quartile 4 (high) | 147/422 | 51.5 | 1.26 (0.96–1.65) | 0.09 |  | 1.27 (0.96–1.66) | 0.09 |  | 0.95 (0.72–1.27) | 0.75 |
| *P* for trend |  |  |  | 0.10 |  |  | 0.08 |  |  | 0.77 |
| *CI* confidence interval, *HR* hazard ratio.  Model 1 adjusted for age, sex, and accelerometer wear time. | | |  |  |  |  |  |  |  |  |
| Model 2 adjusted for education, living alone, body mass index, multimorbidity, fall experience in the past year, low walking ability, cognitive impairment, smoking, drinking plus factors in Model 1. | | | | | | | | | | |
| Model 3 adjusted for moderate-to-vigorous physical activity plus factors in Model 2. | | | | | | | | | |  |
| The quartile cut points were: total sedentary time, 379.1, 455.9, and 537.8 min/day; mean sedentary bout duration, 6.1, 7.5, and 9.4 min/day. | | | | | | | | | | |
