# Supplementary material for: Accelerometer-measured sedentary behavior and risk of functional disability in older Japanese adults: a 9-year prospective cohort study
Source: Int J Behav Nutr Phys Act. 2023 Jul 26;20:91. doi: 10.1186/s12966-023-01490-6 (PMC10369703; doi:10.1186/s12966-023-01490-6)
Supplement: Supplementary file 6 — Additional file 6. Hazard ratios for the risk of functional disability by total sedentary time and mean sedentary bout duration quartiles after excluding participants certified as functional disability in the first two year of follow-up (n = 1,574). [file 12966_2023_1490_MOESM6_ESM.docx]

| **Additional File 6.** Hazard ratios for the risk of functional disability by total sedentary time and mean sedentary bout duration quartiles after excluding participants certified as functional disability in the first two year of follow-up (n = 1,574)^a^ | | | | | | | | | | |
| --- | --- | --- | --- | --- | --- | --- | --- | --- | --- | --- |
|  | No. of events/  participants | Incidence rate  per 1000  person-years | Model 1 | |  | Model 2 | |  | Model 3 | |
|  |  |  | HR (95% CI) | *P* value |  | HR (95% CI) | *P* value |  | HR (95% CI) | *P* value |
| Total sedentary time | |  |  |  |  |  |  |  |  |  |
| Quartile 1 (low) | 77/393 | 24.6 | 1.00 |  |  | 1.00 |  |  | 1.00 |  |
| Quartile 2 | 91/394 | 29.8 | 1.13 (0.84–1.54) | 0.42 |  | 1.14 (0.84–1.55) | 0.41 |  | 0.88 (0.64–1.21) | 0.44 |
| Quartile 3 | 111/393 | 37.2 | 1.28 (0.95–1.72) | 0.11 |  | 1.32 (0.98–1.78) | 0.07 |  | 0.91 (0.66–1.26) | 0.57 |
| Quartile 4 (high) | 122/394 | 42.4 | 1.32 (0.97–1.78) | 0.07 |  | 1.31 (0.96–1.79) | 0.08 |  | 0.78 (0.55–1.12) | 0.18 |
| *P* for trend |  |  |  | 0.057 |  |  | 0.06 |  |  | 0.40 |
| Mean sedentary bout duration | |  |  |  |  |  |  |  |  |  |
| Quartile 1 (low) | 89/409 | 27.9 | 1.00 |  |  | 1.00 |  |  | 1.00 |  |
| Quartile 2 | 95/400 | 30.8 | 1.04 (0.78–1.39) | 0.77 |  | 1.06 (0.79–1.42) | 0.70 |  | 0.94 (0.70–1.26) | 0.69 |
| Quartile 3 | 98/387 | 32.9 | 0.98 (0.73–1.30) | 0.87 |  | 1.01 (0.75–1.35) | 0.95 |  | 0.86 (0.64–1.15) | 0.30 |
| Quartile 4 (high) | 119/378 | 42.4 | 1.11 (0.83–1.47) | 0.48 |  | 1.12 (0.84–1.50) | 0.44 |  | 0.86 (0.63–1.16) | 0.31 |
| *P* for trend |  |  |  | 0.58 |  |  | 0.52 |  |  | 0.26 |

*CI* confidence interval, *HR* hazard ratio.

Model 1 adjusted for age and sex.

Model 2 adjusted for education, living alone, body mass index, multimorbidity, fall experience in the past year, low walking ability, cognitive impairment, smoking, and drinking plus factors in Model 1.

The quartile cut points were as follows: total sedentary time, 457.8, 536.2, and 609.1 min/day; mean sedentary bout duration 6.0, 7.4, and 9.3 min/day.

Model 3 additionally adjusted for moderate-to-vigorous physical activity plus factors in Model 2.

^a^ Values of total sedentary time were corrected for accelerometer wear time by standardizing the total sedentary time to 16 hours per day of accelerometer wear time using the residuals obtained by regressing total sedentary time on accelerometer wear time.
