# Supplementary material for: Accelerometer-measured sedentary behavior and risk of functional disability in older Japanese adults: a 9-year prospective cohort study
Source: Int J Behav Nutr Phys Act. 2023 Jul 26;20:91. doi: 10.1186/s12966-023-01490-6 (PMC10369703; doi:10.1186/s12966-023-01490-6)
Supplement: Supplementary file 8 — Additional file 8. Hazard ratios for the risk of functional disability by total sedentary time and mean sedentary bout duration quartiles after excluding participants who did not normally walk continuously for 15 min at baseline (n = 1,475). [file 12966_2023_1490_MOESM8_ESM.docx]

| **Additional File 8.** Hazard ratios for the risk of functional disability by total sedentary time and mean sedentary bout duration quartiles after excluding participants who did not normally walk continuously for 15 minutes at baseline (n = 1,475) ^a^ | | | | | | | | | | |
| --- | --- | --- | --- | --- | --- | --- | --- | --- | --- | --- |
|  | No. of events/  participants | Incidence rate  per 1000  person-years | Model 1 | |  | Model 2 | |  | Model 3 | |
|  |  |  | HR (95% CI) | *P* value |  | HR (95% CI) | *P* value |  | HR (95% CI) | *P* value |
| Total sedentary time |  |  |  |  |  |  |  |  |  |  |
| Quartile 1 (low) | 75/368 | 25.9 | 1.00 |  |  | 1.00 |  |  | 1.00 |  |
| Quartile 2 | 87/369 | 31.9 | 1.20 (0.88–1.63) | 0.26 |  | 1.27 (0.93–1.74) | 0.13 |  | 1.01 (0.73–1.40) | 0.94 |
| Quartile 3 | 107/369 | 40.3 | 1.37 (1.01–1.84) | 0.04 |  | 1.43 (1.06–1.93) | 0.02 |  | 1.02 (0.74–1.41) | 0.91 |
| Quartile 4 (high) | 122/369 | 48.7 | 1.44 (1.06–1.96) | 0.02 |  | 1.48 (1.08–2.01) | 0.01 |  | 0.93 (0.65–1.32) | 0.67 |
| *P* for trend |  |  |  | 0.02 |  |  | 0.01 |  |  | 0.65 |
| Mean sedentary bout duration | |  |  |  |  |  |  |  |  |  |
| Quartile 1 (low) | 82/369 | 29.0 | 1.00 |  |  | 1.00 |  |  | 1.00 |  |
| Quartile 2 | 88/374 | 31.7 | 1.00 (0.74–1.35) | 0.99 |  | 1.00 (0.74–1.36) | 0.99 |  | 0.89 (0.66–1.22) | 0.47 |
| Quartile 3 | 101/377 | 37.0 | 1.06 (0.79–1.43) | 0.68 |  | 1.15 (0.85–1.54) | 0.37 |  | 0.97 (0.71–1.31) | 0.82 |
| Quartile 4 (high) | 120/355 | 48.9 | 1.18 (0.88–1.58) | 0.27 |  | 1.20 (0.89–1.61) | 0.24 |  | 0.92 (0.67–1.25) | 0.60 |
| *P* for trend |  |  |  | 0.23 |  |  | 0.15 |  |  | 0.76 |
| *CI* confidence interval, *HR* hazard ratio.  Model 1 adjusted for age and sex. | | | | | | | | | | |
| Model 2 adjusted for education, living alone, body mass index, multimorbidity, fall experience in the past year, cognitive impairment, smoking, drinking plus factors in Model 1. | | | | | | | | | | |
| Model 3 adjusted for moderate-to-vigorous physical activity plus factors in Model 2. | | | | | | | | | | |
| The quartile cut points were: total sedentary time, 457.4, 535.9, and 606.3 min/day; mean sedentary bout duration 6.1, 7.5, and 9.3 min/day. | | | | | | | | | | |
| ^a^ Total sedentary time was adjusted for wearing time using the residuals method. | | | | | | |  |  |  |  |
