# Supplementary material for: Accelerometer-measured sedentary behavior and risk of functional disability in older Japanese adults: a 9-year prospective cohort study
Source: Int J Behav Nutr Phys Act. 2023 Jul 26;20:91. doi: 10.1186/s12966-023-01490-6 (PMC10369703; doi:10.1186/s12966-023-01490-6)
Supplement: Supplementary file 9 — Additional file 9. Multivariable adjusted hazard ratios of functional disability associated with 10-min changes in time spent in sedentary, LPA, and MVPA after excluding participants who did not normally walk continuously for 15 min at baseline (n = 1,475). [file 12966_2023_1490_MOESM9_ESM.docx]

| **Additional File 9.** Multivariable adjusted hazard ratios of functional disability associated with 10-min changes in time spent in sedentary, LPA, and MVPA after excluding participants who did not normally walk continuously for 15 minutes at baseline (n = 1,475)^a^ | | | | | |
| --- | --- | --- | --- | --- | --- |
|  | With sedentary time |  | With LPA |  | with MVPA |
|  | HR (95% CI) |  | HR (95% CI) |  | HR (95% CI) |
| Replace sedentary time | ‒ |  | 1.00 (0.99‒1.01) |  | 0.89 (0.84‒0.93) |
| Replace LPA | 1.00 (0.99‒1.01) |  | ‒ |  | 0.88 (0.84‒0.93) |
| Replace MVPA | 1.13 (1.08‒1.19) |  | 1.13 (1.07‒1.19) |  | ‒ |
| *CI* confidence interval, *HR* hazard ratio, *LPA* light physical activity, *MVPA* moderate-to-vigorous physical activity.  Models were adjusted for sex, age, living alone, body mass index, multimorbidity, fall experience in the past year, cognitive impairment, smoking, drinking, and wearing time.  ^a^ Total sedentary time were corrected for accelerometer wear time by standardizing the total sedentary time to 16 hours per day of accelerometer wear time using the residuals obtained by regressing total sedentary time on accelerometer wear time. | | | | | |
